# Tertiary cystic white matter injury as a potential phenomenon after hypoxia-ischaemia in preterm f sheep

**DOI:** 10.1093/braincomms/fcab024

**Published:** 2021-03-09

**Authors:** Benjamin A Lear, Christopher A Lear, Joanne O Davidson, Jialin Sae-Jiw, Johanna M Lloyd, Alistair J Gunn, Laura Bennet

**Affiliations:** The Fetal Physiology and Neuroscience Group, Department of Physiology, The University of Auckland, Auckland 1142, New Zealand

**Keywords:** hypoxia-ischaemia, preterm, periventricular leukomalacia, tertiary injury, cystic white matter injury

## Abstract

White matter injury, including both diffuse and cystic elements, remains highly associated with neurodevelopmental disability and cerebral palsy in preterm infants, yet its pathogenesis and evolution are still poorly understood and there is no established treatment. We examined the long-term evolution of white matter injury in chronically instrumented preterm fetal sheep (0.7 gestation) after 25 min of complete umbilical cord occlusion or sham occlusion. Fetal brains were processed for histology after 3 days (*n* = 9, sham *n* = 9), 7 days (*n* = 8, sham *n* = 8), 14 days (*n* = 9, sham *n* = 8) and 21 days (*n* = 9, sham *n* = 9) of recovery. At 3 and 7 days recovery, umbilical cord occlusion was associated with diffuse white matter injury, with loss of total and mature oligodendrocytes and reduced myelination in both the parietal and temporal lobes. At 14 days after umbilical cord occlusion, extensive microglial and astrocytic activation were observed in the temporal lobe. At 21 days recovery a spectrum of severe white matter degeneration was observed, including white matter atrophy, ventriculomegaly and overt cystic white matter lesions. The most severe injury was observed in the temporal lobe after 21 days recovery, including the majority of cystic lesions, persistent oligodendrocyte maturational arrest and impaired myelination. The spatial distribution of delayed white matter degeneration at 21 days recovery was closely related to the location of dense microglial aggregates at earlier time-points, implicating a role for exuberant inflammation originating from microglial aggregates in the pathogenesis of cystic white matter injury. The delayed appearance of cystic injury is consistent with continuing tertiary evolution of necrotic cell death. This slow evolution raises the tantalizing possibility that there may a relatively long therapeutic window to mitigate the development of cystic white matter injury. Delayed anti-inflammatory treatments may therefore represent a promising strategy to reduce neurodevelopmental disability in the preterm infants.

## Teaser

Cystic white matter injury is the key pathological substrate of cerebral palsy in preterm infants. Lear et al. show that in fetal sheep cystic injury after severe hypoxia evolved over many weeks, raising the tantalizing possibility that substantial white matter protection may be possible with very delayed interventions.

## Graphical Abstract

**Figure:**
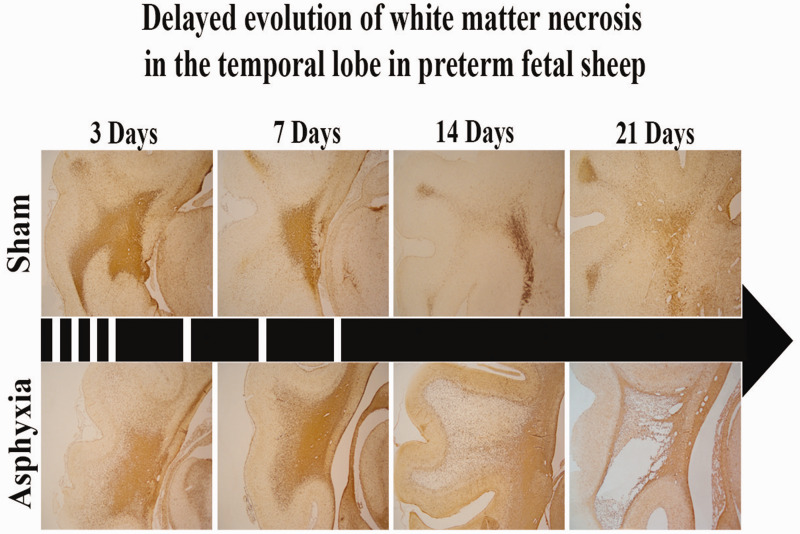

## Introduction

Preterm infants have very high rates of neurodevelopmental disability, including over one-third of all cases of cerebral palsy.^1^ Although the incidence of cerebral palsy may have fallen slightly in some populations, absolute rates remain high and overall neurodevelopmental outcomes have not clearly changed despite improvements in clinical care.^2^ Structurally, cerebral palsy is associated with severe cystic white matter injury (WMI), previously called cystic periventricular leukomalacia.^3–5^ It is now well established that milder injury leads to astrogliosis, maturational arrest of oligodendrocytes and impaired myelination.^6–8^ Although rates of cystic-WMI have gradually fallen,^9^ cystic-WMI remains highly associated with severe disability and cerebral palsy.^10^^,^^11^ Contemporary post-mortem cohort studies suggest that large necrotic lesions were found in about one-third of cases.^12^ Thus, it remains important to understand the factors contributing to cystic-WMI in order to reduce the incidence of cerebral palsy. 

There is no established treatment for preterm brain injury. Hypoxia-ischaemia (HI) before or during birth is a major contributor to neurodevelopmental disability, and disproportionately affects preterm-born infants.^6^^,^^13^^,^^14^ Experimentally, after acute HI in the immature brain, extensive cell loss most often occurs during the first few days, concomitant with secondary loss of cerebral oxidative metabolism.^15^ However, in some studies injury continued to evolve over days and weeks during a tertiary phase.^16^ Clinically, cystic-WMI is most often seen on ultrasonography at a median of ∼4 weeks after birth,^17^ although more severe cysts tended to appear earlier.^11^^,^^17^ This apparent delay suggests that injury may be evolving over many weeks in the tertiary phase. The mechanisms of this tertiary cell loss are not fully understood. Thus, to develop targeted therapies, better understanding of how preterm brain injury evolves is needed.

Studies in preterm fetal sheep have typically evaluated evolving injury over 3−7 days post-HI, demonstrating a staged evolution of both white and grey matter injury, reparative proliferation, microglial induction and impaired oligodendrocyte development,^18–21^ consistent with contemporary post-mortem findings.^5^^,^^8^^,^^12^^,^^22^ More limited data at 14 days recovery showed a significant reduction in the total number of neurons and oligodendrocytes, along with dysmaturation of somatostatin neurons,^19^^,^^23^ consistent with findings in preterm infants.^24^ Furthermore, 21 days after severe HI, there was selective loss of neurons and white matter in selected regions, associated with chronic inflammation.^25^ However, there is little systematic, regional information on how WMI evolves.

We therefore undertook an hypothesis-generating study of the regional evolution of WMI at 3, 7, 14 and 21 days after umbilical cord occlusion (UCO). In sheep this period broadly spans brain maturation from 30 weeks to term.^26^

## Materials and methods

### Ethical approval

Ethical approval was obtained from the Animal Ethics Committee of the University of Auckland, and all procedures were carried out in accordance with the New Zealand Animal Welfare Act 1999 and the University of Auckland’s Code of Ethical Conduct for the use of animals for teaching and research, approved by the Ministry of Primary Industries, Government of New Zealand. This manuscript is compliant with the ‘Animal Research: Reporting of In Vivo Experiments’ guidelines.^27^

### Surgical procedures

A total 69 Romney/Suffolk fetal sheep were surgically instrumented at 98–100 days of gestation (term is 147 days). Ewes were given antibiotic prophylaxis intramuscularly 30 min before surgery (20 mg/kg oxytetracycline, Phoenix Pharm Distributors, Auckland, New Zealand). Anaesthesia was induced by intravenous propofol (5 mg/kg, AstraZeneca, Auckland, New Zealand) and maintained by 2–3% isoflurane in oxygen.

Surgical procedures have been described previously.^28^^,^^29^ In brief, the foetus was partially exteriorized and polyvinyl catheters (SteriHealth, Dandenong South, VIC, Australia) were placed in the femoral and brachial arteries and the amniotic space. ECG electrodes were placed (Cooner Wire, Chatsworth, CA, USA) and an inflatable silicone occluder (OC18HD, In Vivo Metric, Healdsburg, CA, USA) was fitted around the umbilical cord. Fetal leads were exteriorized through the maternal flank, and a maternal long saphenous vein was catheterised. Gentamicin was administered into the amniotic space (80 mg, Pfizer, Auckland, New Zealand) and the maternal skin incision was infiltrated with a long-acting local analgesic (0.5% bupivacaine plus adrenaline, AstraZeneca).

### Post-operative care

Ewes were housed together in separate metabolic cages under controlled environmental conditions (16 ± 1°C, 50 ± 10% humidity, lights on 0600–1800 h). Ewes had *ad libitum* access to food and water and were given 4 days of intravenous antibiotics (600 mg benzylpenicillin sodium, Novartis, Auckland, New Zealand; 80 mg gentamicin, Pfizer). Fetal catheters were placed on continuous heparinized saline infusions to maintain patency (20 U/ml at 0.2 ml/h).

### Data acquisition and recordings

Physiological signals were recorded continuously using customized LabVIEW-based data acquisition software (National Instruments, Austin, TX, USA); details of signal processing methods have been previously reported.^28^^,^^29^ Fetal arterial blood pressure was corrected for maternal position by subtraction of amniotic fluid pressure (Novatrans III Gold MX860 pressure transducers, Medex, Hilliard, OH, USA).

### Experimental protocol

Experiments began at 9:00 am, 4–6 days after surgery (104–105 days of gestation). Foetuses were randomly assigned to receive HI induced by complete UCO or sham-UCO (sham). UCO was performed for 25 min, but was ended early if severe hypotension occurred (mean arterial pressure, MAP <8 mmHg).^28^ Sham foetuses received no UCO. Foetuses were allowed to recover for 3 days [3d-UCO *n* = 9 (5 Female : 4 Male), 3d-sham *n* = 9 (5 F : 4 M)], 7 days [7d-UCO *n* = 8 (4 F : 4 M), 7d-sham *n* = 8 (3 F : 5 M)], 14 days [14d-UCO *n* = 8 (6 F : 2 M), 14d-sham *n* = 9 (6 F : 3 M)] or 21 days [21d-UCO *n* = 9 (6 F : 3 M), 21d-sham *n* = 9 (4 F : 5 M)] after UCO. At the end of their respective recovery periods, ewes and foetuses were killed by an overdose of sodium pentobarbitone given intravenously to the ewe (9 g Pentobarb 300, Provet New Zealand, Auckland, New Zealand). This method is consistent with the Animal Welfare Act of New Zealand.

### Arterial blood samples

Fetal arterial blood samples (0.3 ml) were taken before the start of the experiment, at 5 and 17 min during occlusion, and at 2, 4 and 6 h after occlusion, then daily thereafter between 8:30 and 9:30 am. Blood samples were analysed for pH and blood gases (ABL 800, Radiometer, Copenhagen, Denmark) and glucose and lactate levels (YSI model 2300, Yellow Springs, OH, USA). Blood gases were taken from all groups, but only those from the 21d-sham and 21d-UCO groups are presented for clarity.

### Histological preparation

At post-mortem, fetal brains were perfusion fixed *in situ* with 10% phosphate-buffered formalin. Brains were further emersion fixed for 1 week before being processed and paraffin embedded. Coronal sections (10 µm thickness) were cut with a rotary microtome (RM2235, Leica Microsystems, Wetzlar, Germany). Sections containing the regions of interest for the present study were selected from 17 mm anterior to stereotaxic zero.^30^ Sections were dewaxed in xylene, rehydrated in decreasing concentrations of ethanol and washed in phosphate buffered saline. Sections were stained either with thionine (Scharlau, Barcelona, Spain) plus acid fuchsin (Sigma-Aldrich, Sydney, NSW, Australia) to examine macroscopic structural integrity or for immunohistochemistry.

For immunohistochemistry, antigen retrieval was performed in citrate buffer with the 2100 Antigen Retriever (Aptum Biologics, Southampton, UK). Endogenous peroxidase was quenched by incubation in 1% hydrogen peroxide in methanol [or in phosphate buffered serum, for oligodendrocyte transcription factor-2 (Olig-2)]. Blocking was performed for 1 h at room temperature in 3% normal goat serum. Sections were labelled with monoclonal primary antibodies at 1:200 concentration in 3% normal goat serum overnight at 4°C: Rabbit anti-Olig-2 (Merck-Millipore, Bellerica, MA, USA), mouse anti‐adenomatous polyposis coli (hence forth referred to as CCI, Merk-Millipore), mouse anti-2',3'-cyclic-nucleotide 3'-phosphodiesterase (CNPase, Merck-Millipore), rabbit anti-myelin basic protein (MBP, Merck-Millipore), goat anti-ionized calcium binding adaptor molecule-1 (Iba-1, Abcam), rabbit anti-glial fibrillary acidic protein (GFAP, Abcam) and rabbit anti-caspase-3 (Abcam). Sections were incubated for 3 h with the species appropriate biotin-conjugated monoclonal secondary antibody (Vector Laboratories, Burlingame, CA, USA) at 1:200 dilution in 3% normal goat serum. Slides were incubated in ExtrAvidin (Sigma-Aldrich) at a dilution of 1:200 in 3% normal goat serum for 2 h and then reacted with diaminobenzidine tetrachloride (Sigma-Aldrich). The reaction was halted by immersion in phosphate buffered saline. Slides stained with acid fuchsin/thionine or immunohistochemistry were dehydrated in increasing concentrations of ethanol followed by xylene and finally mounted with coverslips. Two sections were analysed for each stain/antibody.

### White matter and cystic lesion area analysis

Macroscopic examination of the structural integrity of the parietal and temporal lobes white matter was performed on sections stained with acid fuchsin/thionine using light microscopy at 2−10× magnification by a blinded assessor using an Eclipse 80i microscope (Nikon, Tokyo, Japan).

Whole section images were also taken on a Zeiss Axio Imager Z2 microscope with automated motorized stage (Carl Zeiss AG, Oberkochen, Germany). Serial images were collected at 2.5× magnification and collated using VSlide stitching software (MetaSystems, Altlussheim, Germany). Using these images, total white matter and ventricle area were measured using ImageJ software (National Institute of Health, Bethesda, MD, USA). Total white matter area was measured across all continuous white matter structures of the parietal and temporal lobes of both hemispheres, including the intragyral and periventricular regions and the corpus callosum. Macroscopic cystic white matter lesions were identified in the 21d-UCO group, most commonly in the temporal lobe. The area of each identified lesion was measured, and this area was subtracted from the total white matter area to give a final intact white matter area. For the purpose of this analysis, cystic tissue was defined as macroscopically evident irregular architecture or cellular loss.^31^

### Temporal and parietal lobe microscopy

**Figure 1:**
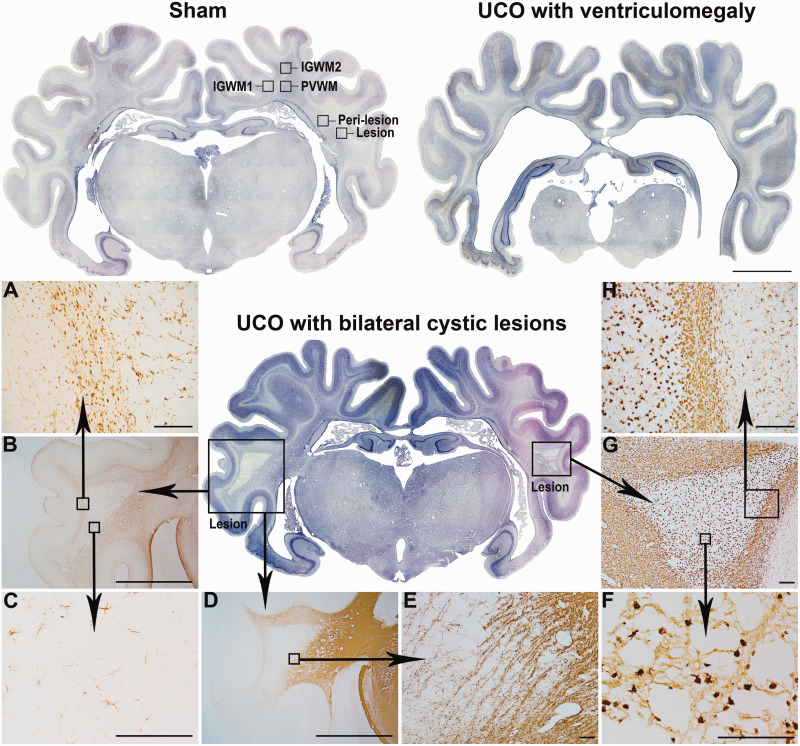
**Spectrum of macroscopic white matter degeneration at 21 days. Examples of whole coronal sections after 21 days of recovery in the sham group with regions of interest labelled (top left) and the two predominant patterns of macroscopic WMI in the 21d-UCO group: White matter atrophy with ventriculomegaly (top right) and bilateral cystic white matter lesions (centre) which were primarily observed in the temporal lobe white matter.** Serial images were taken at ×2.5 magnification and stitched together (scale bar = 1000 µm). (**A**−**H**) Representative higher magnification photomicrographs of the cystic lesions. (**A**−**C**) (Taken at ×10, ×2.5, ×20, respectively) show GFAP-positive astrocytes illustrating that a dense border of astrogliosis formed around the perimeter of the lesions (**A**) while a marked reduction in the numbers of astrocytes was observed within the centre of the lesion (**C**). (**D**−**E**) (Taken at ×2.5 and ×4, respectively) show that MBP-positive myelin was severely disrupted within and surrounding the lesion. (**F**−**H**) (Taken at ×40, ×4 and ×10, respectively) show Iba-1-positive microglia with darkly stained amoeboid morphologies in the centre of the lesion (**F**) and a dense border of amoeboid microglia surrounding the perimeter of the lesion (**H**). (Scale bar = 1000 µm for ×2.5, 200 µm for ×4, ×10 and ×20 magnifications, and 100 µm for ×40 magnification).

Images of tissue labelled for oligodendrocytes (Olig-2-positve cells), mature oligodendrocytes (CC1-positive cells), myelin (MBP and CNPase-positive fibres), microglia (Iba-1 positive cells), astrocytes (GFAP-positive cells), and apoptotic cells (caspase-3-positive cells) were taken from two regions in the temporal lobe and three regions in the parietal lobe (Fig. 1) at 20× magnification by an assessor blinded to the group by independent coding of slides and data files on an Eclipse 80i microscope (Nikon). These regions of interest are shown in Fig. 1.

In the temporal lobe, images were taken from the centre of the temporal lobe white matter (consistent with the centre of the cystic lesions identified in the 21d-UCO group) in order to capture the evolution of the cystic lesions. Due to the presence of cystic lesions at 21 days, these images were only taken in the 3d, 7d and 14d groups. A second set of images were taken from a peri-lesion region in order to assess temporal white matter integrity surrounding the cystic lesions. This region was defined by the position and size of the lesions in the 21d-UCO group and positioned adjacent to the superior-medial border of the lesions, outside the border of Iba-1-positive and GFAP-positive cells surrounding the lesion for each individual animal. Images from representative regions were taken in animals that had no identifiable cystic lesions, including earlier time-points and sham animals. In the parietal lobe, images were taken from the first and second intragyral white matter (IGWM1, IGWM2) and the periventricular white matter regions (PVWM). Images for the three parietal lobe regions and the peri-lesion region were taken at all time-points. The locations that images were taken from in both the parietal and temporal lobe are shown in Fig. 1.

All analyses were performed by a blinded assessor. Cell counts were performed manually using ImageJ (National Institute of Health). Microglia showing either an amoeboid or ramified morphology were included. The area fractions of CNPase and MBP were quantified using ImageJ set at the default thresholding (National Institute of Health).

A heat map displaying the location and frequency of microglial aggregates present in 3d-sham animals was created using images of the left hemisphere of coronal sections stained for Iba-1 positive microglia. The area of microglial aggregates from each animal were traced using ImageJ (National Institute of Health) before being combined as a z-stack. A summation of overlapping areas created a range of intensities, which were coloured to represent the number of overlapping microglial aggregates in the z-stack. These intensities were finally overlaid on an unaltered image of a coronal section labelled with Iba-1.

### Statistical analysis

Statistical analysis was performed using SPSS v25 (SPSS, Chicago, IL, USA). The sample size was selected based on oligodendrocyte survival. With an estimated pooled standard deviation of 90 from previous studies, an *n* = 8 or higher offered 90% power to detect a 20% or greater loss of oligodendrocytes after UCO compared with sham-UCO. Histological outcomes in the three parietal lobe regions (IGWM1/2 and PVWM) were assessed using three-way analysis of variance (ANOVA) with group as the independent factor and area and time treated as repeated measures. Histological outcomes in the two temporal lobe regions were assessed separately using two-way ANVOA with group as the independent factor and time as a repeated factor. Individual time-points were tested *posthoc* if a significant effect of time was found via two-way or one-way ANOVA for the parietal and temporal lobes, respectively. Post-mortem weights were assessed by two-way ANOVA with group as the independent factor and time as a repeated measure. Biochemistry outcomes were assessed by one-way ANOVA with group as the independent factor. Statistical significance was accepted when *P* < 0.05. Data are presented as mean ± SEM.

### Data availability

Original data are available from the authors on reasonable request.

## Results

### Baseline and UCO

All foetuses across all cohorts were healthy before the start of experiments including normal physiological and arterial blood gas parameters by our laboratory standards. There were no significant differences in any physiological parameters between the groups before experiments. UCO was associated with sustained bradycardia and profound arterial hypotension, hypoxemia, hypoglycaemia and progressive respiratory and metabolic acidosis (Table 1). There were no differences in the duration of occlusions or the severity of hypotension at the end of occlusion between the HI groups (3d-UCO 12.1 ± 1.3, 7d-UCO 14.1 ± 1.5, 14d-UCO 11.9 ± 0.9, 21d-UCO 12.1 ± 0.9 mmHg). Fetal heart rate and MAP recovered rapidly after the end of occlusion. Fetal demographics and post-mortem outcomes are presented in Table 2.

### Delayed macroscopic WMI and cystic lesions

**Figure 2:**
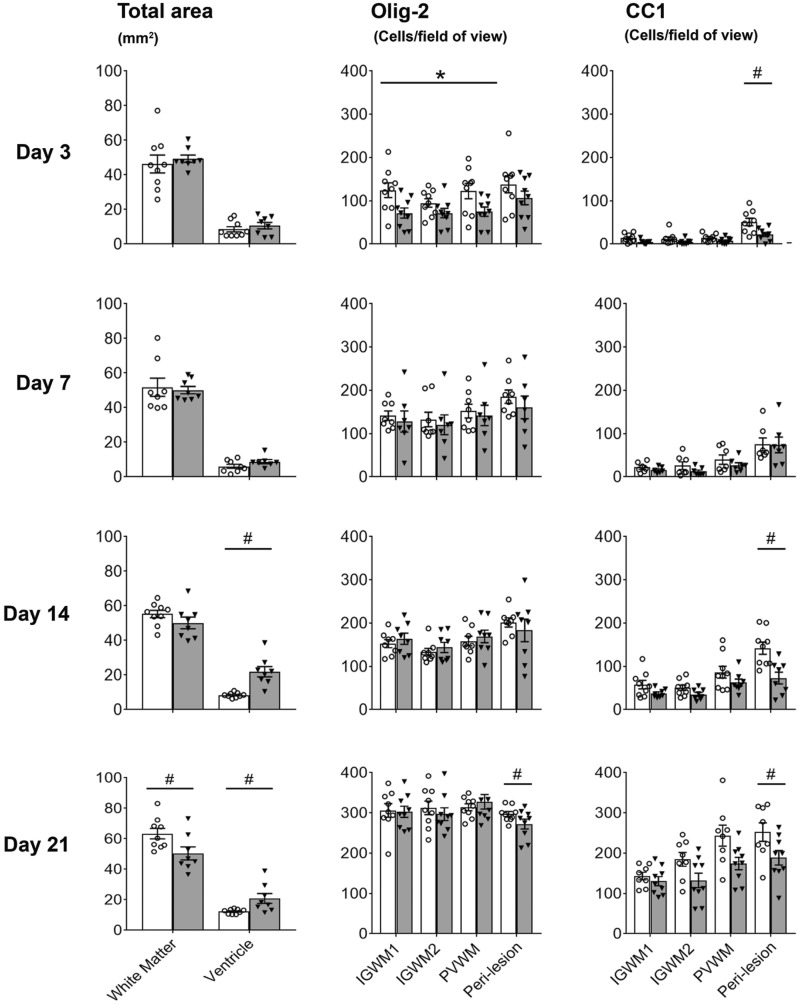
**Area of white matter and lateral ventricles, and numbers of Olig-2 and CC1-positive oligodendrocytes.** Left panels: total white matter and ventricle areas. Middle panels: Numbers of total (Olig-2) oligodendrocytes in the parietal (IGWM1/2, PVWM) and temporal (peri-lesion) lobes. Right panels: Numbers of mature (CC1) oligodendrocytes in the parietal (IGWM1/2, PVWM) and temporal (peri-lesion) lobes. Sham occlusion is shown in white columns, UCO is shown in grey columns. Three-way ANOVA including time and the three regions (IGWM1/2 and PVWM) as repeated measures showed a significant reduction in CC1 positive oligodendrocytes in the UCO groups in the parietal lobe regions (*P* < 0.05). Two-way ANOVA including time as a repeated measure showed a significant reduction in CC1 and Olig-2-positive oligodendrocytes in the UCO groups within the peri-lesion region of the temporal lobe (*P* < 0.05). Statistical significance shown on the figure was determined on individual time-points by one-way ANOVA for total areas and the peri-lesion region (^#^*P* < 0.05 sham versus UCO) and two-way ANOVA for the parietal lobe with area (IGWM1/2 and PVWM) as a repeated measure (*P < 0.05 sham versus UCO). Data are means ± SEM. N=Sham/UCO, total white matter and lateral ventricle area: 3d 9/8, 7d 8/8, 14d 9/8, 21d 9/8. Olig-2: 3d 9/9, 7d 8/7, 14d 8/8, 21d 9/9. CC1: 3d 9/9, 7d 7/7, 14d 9/8, 21d 8/9.

A spectrum of macroscopic WMI was observed in the 21d-UCO group, as shown in Fig. 1 and summarized in Table 3. In brief, 4/9 foetuses in the 21d-UCO group developed marked white matter atrophy and ventriculomegaly. Cystic white matter lesions were observed in an overlapping group of 4/9 foetuses; only 2 foetuses did not show either cystic lesions or ventriculomegaly. Across the entire 21d-UCO group, white matter area was significantly reduced (21d-sham 63.3 ± 3.4 versus 21d-UCO 50.4 ± 4.3 mm^2^, *P* < 0.05, Fig. 2) and lateral ventricle area was significantly increased (21d-sham 12.3 ± 0.5 versus 21d-UCO 20.8 ± 3.3 mm^2^, *P* < 0.05, Fig. 2). The white matter of the temporal lobe was the most frequently and severely affected by cystic white matter lesions, including two foetuses that showed near complete destruction of all white matter in the temporal lobe. The cystic lesions were associated with a marked reduction of all cellular elements (Fig. 1), including low numbers of oligodendrocytes (Olig-2- and CC1-positive) and myelin proteins (CNPase- and MBP-positive) and were sparsely populated with densely labelled Iba-1-positive amoeboid microglia. GFAP-positive astrocytes were dysmorphic, with fragmented or absent processes. A dense border of amoeboid Iba-1-positive microglia and GFAP-positive astrocytes formed around the periphery of the lesions (Fig. 1).

In contrast, no established cystic lesions were observed in the 3d-UCO, 7d-UCO or 14d-UCO groups. In the 3d-UCO and 7d-UCO groups, no differences were observed in white matter area (3d-sham 46.2 ± 5.1 versus 3d-UCO 49.4 ± 2.1 mm^2^, 7d-sham 49.3 ± 9.1 versus 7d-UCO 50.1 ± 2.1 mm^2^; Fig. 2) or lateral ventricle area (3d-sham 8.5 ± 1.5 versus 3d-UCO 10.7.4 ± 2.2 mm^2^, 7d-sham 6.1 ± 1.2 versus 7d-UCO 8.8 ± 1.2 mm^2^). In the 14d-UCO group, no change in white matter area was observed (14d-sham 55.3 ± 2.2 versus 14d-UCO 50.1 ± 3.4 mm^2^, Fig. 2) but a significant increase in lateral ventricle area was observed (14d-sham 12.1 ± 2.4 versus 14d-UCO 21.9 ± 3.0 mm^2^, *P* < 0.05, Fig. 2).

### Macroscopic WMI was preceded by intense white matter inflammation

**Figure 3:**
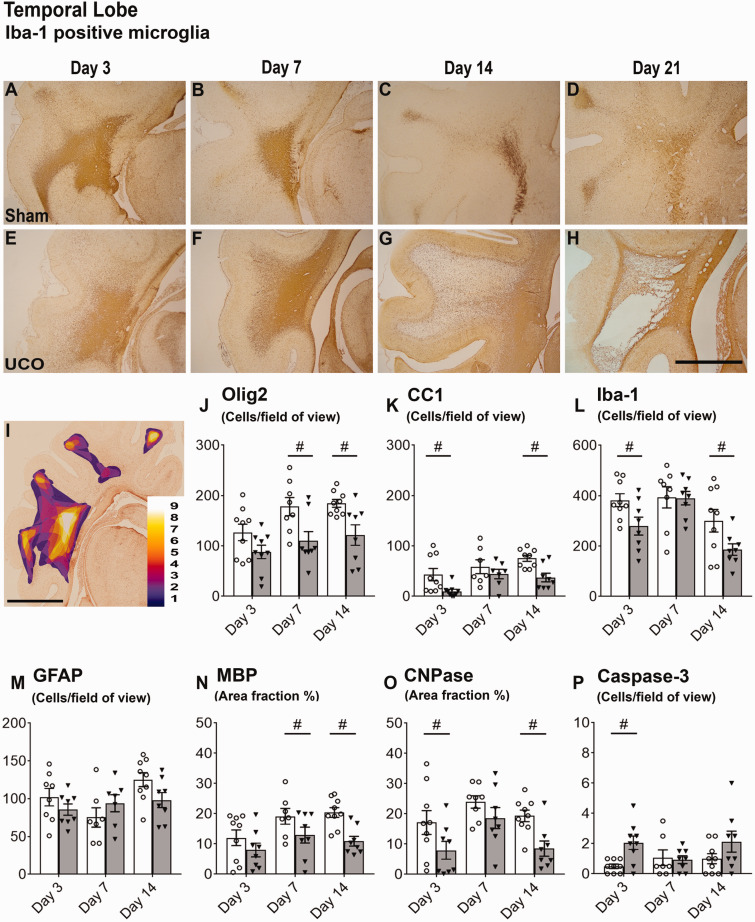
**Evolution of cystic lesions and histological outcomes in temporal white matter.** Top panels: evolution of cystic lesions in the temporal lobe (**A**−**D** sham, **E**−**H** umbilical cord occlusion, UCO). Images are coronal sections stained with Iba-1 taken at ×2.5 magnification (scale bar = 1000 µm). Note the large microglial aggregates in the 3d-sham and 3d-UCO groups (**A** and **E**) and 7d-sham and 7d-UCO groups (**B** and **F**), the marked dispersal of microglia in the 14d-UCO group (**G**) and the cystic lesion surrounded by a gliotic barrier in the 21d-UCO group (**H**). (**I**) A coronal section stained with Iba-1 from the 3d-sham group showing both the parietal and temporal lobe, overlaid with a heat map demonstrating the location of Iba-1-positive microglial aggregates across the 3d-sham group (scale bar = 1000 µm). Colours correspond to the number of foetuses in the 3d-sham group showing a microglial aggregate in that region. Note the frequent, large microglial aggregate within the temporal white matter. Bottom panels (**J**−**O**): histological outcomes from the centre of the temporal lobe at 3, 7 and 14 days after sham occlusion (white columns) and UCO (grey columns). Two-way ANOVA including time as a repeated measure showed an overall significant reduction in Olig-2 (**J**) and CC1 (**K**) positive oligodendrocytes and CNPase (**N**) positive myelin (*P* < 0.05). Statistical significance shown in the figure was determined on individual time-points by one-way ANOVA (^#^*P* < 0.05 sham versus UCO). Data are means ± SEM. N = Sham/UCO, Olig-2: 3d 9/9, 7d 8/8, 14d 9/8. CC1: 3d 9/9, 7d 7/7, 14d 9/8. Iba-1: 3d 9/8, 7d 8/8, 14d 9/8. GFAP: 3d 8/8, 7d 7/7, 14d 9/8. MBP: 3d 9/8, 7d 7/8, 14d 9/8. CNPase: 3d 9/8, 7d 8/8, 14d 9/8. Caspase-3: 9/9, 7d 7/8, 14d 9/8.

**Figure 4:**
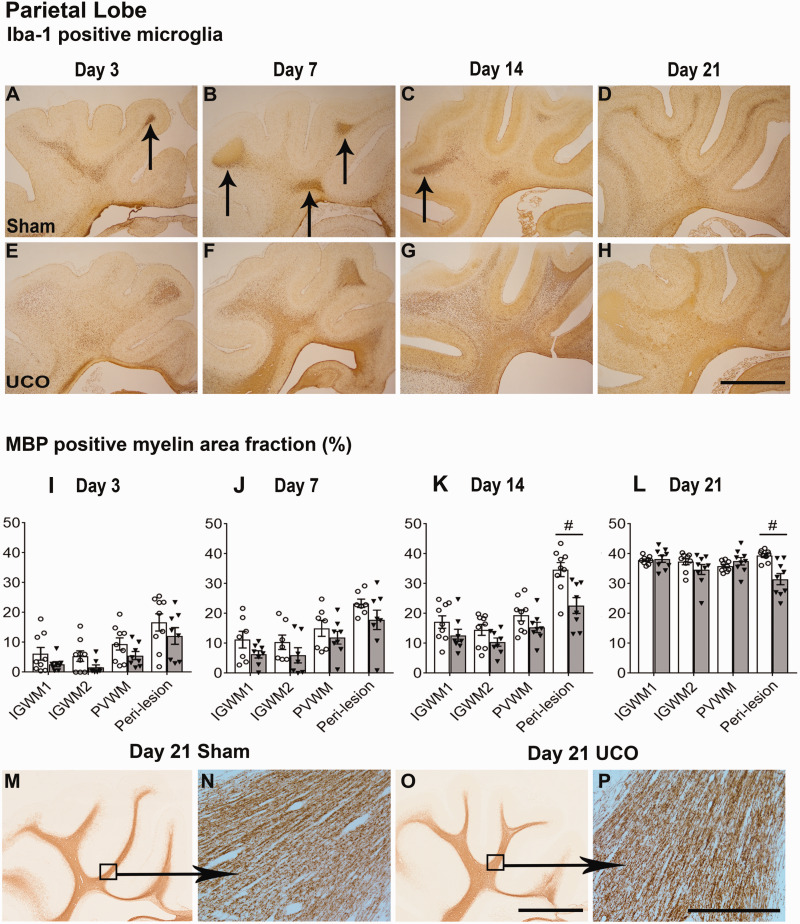
**Parietal white matter histological outcomes. Top panels: Coronal sections showing the parietal lobe stained with Iba-1 taken at ×2.5 magnification (scale bar = 1000 µm).** Note the transient nature of microglial aggregates (arrows) within the sham groups (**A**−**D**) compared with the profound changes seen throughout the entire parietal lobe in the UCO group (**E**−**H**). Middle panels (**I**−**L**): graphs showing changes in myelination quantified as area fraction of MBP positive staining after sham occlusion (white columns) and UCO (grey columns). Three-way ANOVA including time and the three regions (IGWM1/2 and PVWM) as repeated measures showed a significant reduction in MBP positive myelin in the UCO groups within the parietal lobe regions (*P* < 0.05). Two-way ANOVA including time as a repeated measure showed a significant reduction in MBP positive myelin in the UCO groups within the peri-lesion region of the temporal lobe (*P* < 0.05). Statistical significance shown on the figure was determined on individual time-points by one-way ANOVA for the peri-lesion region (^#^*P* < 0.05 sham versus UCO). Data are means ± SEM. N = Sham/UCO, MBP: 3d 7/8, 7d 7/8, 14d 9/8, 21d 9/9. Bottom panels: ×2.5 magnification images (**M**, **O**) of the parietal lobe stained with MBP with representative ×20 images (**N**, **P**). Note that the MBP positive myelin appeared morphologically similar between sham and UCO groups at 21 days (Scale bars = 1000 µm for ×2.5 and 200 µm for ×20 images).

The eventual locations of cystic white matter lesions in the 21d-UCO group appeared to be closely related to the location of large microglial aggregates at earlier time-points, which consisted of high densities of small amoeboid microglia. The largest and most consistent aggregates occupied large proportions of the temporal lobe and were observed in both sham and UCO groups at all time-points (Fig. 3). Smaller aggregates were located in the parietal lobe, at the top of the parasagittal white matter and less consistent small aggregates were observed in the PVWM (Fig. 4). Aggregates in the parietal lobes were observed in the sham and UCO groups at 3−7d but, in contrast to those in the temporal lobe, had nearly all dispersed by 14-21d (Fig. 4).

We contrasted the pattern of macroscopic WMI observed in the 21d-UCO group with the microscopic white matter changes in the 3d-UCO, 7d-UCO and 14d-UCO groups focussing on the temporal lobe white matter and the associated microglial aggregates (Fig. 3). UCO was associated with complex changes to microglia morphology and density within the temporal lobe aggregates. In the 3d-UCO group, microglia were larger, more densely stained and more dispersed resulting in a significant reduction in the density of microglia within the centre of the temporal lobe white matter (*P* < 0.05). In contrast, there were no differences in density and few morphological differences between the 7d-UCO and 7d-sham groups. Marked, but variable changes in microglia were observed in the 14d-UCO group. Overall, microglial density within the temporal lobe aggregate was reduced in the 14d-UCO group (*P* < 0.05, Fig. 3). This was related to the predominant pattern in 4/8 foetuses of large, densely stained amoeboid microglia, similar to those observed within the lesions of the 21d-UCO group. There was a significant reduction of Iba-1 and GFAP positive area fraction within the centre of the temporal lobe white matter (both *P* < 0.05, data not shown). This pattern extended throughout the temporal lobe and was also seen in the parietal lobe in 2/4 of these foetuses. In these above cases, the affected white matter was surrounded by a dense border of GFAP-positive astrocytes. Three out of the eight foetuses instead showed increased density of microglia within the temporal lobe white matter and isolated areas of the parietal lobe. Only 1/8 foetus in the 14d-UCO group did not show any change in microglial morphology or area fraction compared with shams.

Across the three time-points (3, 7 and 14 days), within the centre of the temporal lobe UCO was associated with a significant decrease in numbers of Olig-2 (*P* < 0.05) and CC1-positive (*P* < 0.05) oligodendrocytes and reduced CNPase (*P* < 0.05) and MBP (*P* < 0.05) positive myelin area fraction within the temporal lobe (Fig. 3). However, there was no significant change in the number of GFAP positive astrocytes or caspase-3 positive apoptotic cells. *Posthoc* analysis showed that UCO was associated with a significant reduction in Iba-1 positive microglia cells in the 3d-UCO and 14d-UCO groups (both *P* < 0.05), as well as a significant increase in caspase-3 positive apoptotic cells in the 3d-UCO group (*P* < 0.05).

### The temporal lobe also developed non-cystic diffuse WMI

**Figure 5:**
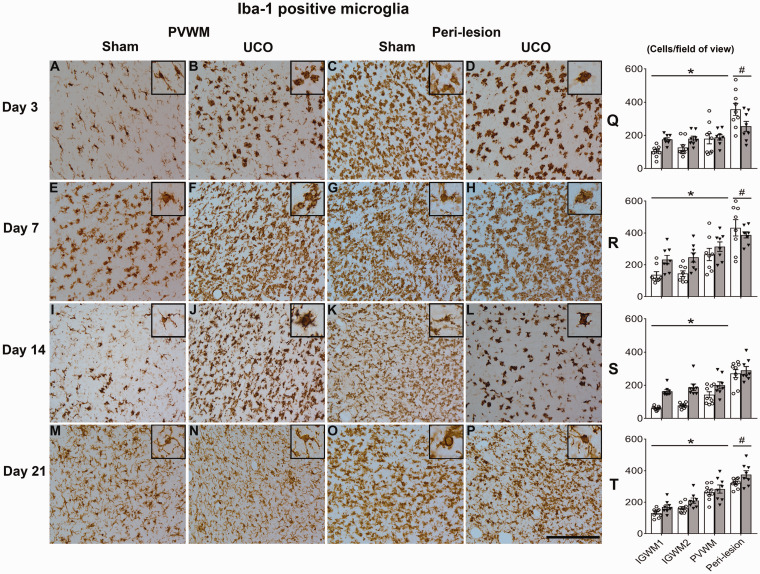
**Microglia in non-cystic regions.** Left panels (**A**−**P**): representative photomicrographs of Iba-1-positive microglia in the PVWM (parietal lobe) and peri-lesion region (temporal lobe). Inserts show higher magnification of microglial morphology. Note the profound morphology and density changes between sham and UCO groups, especially in the peri-lesion region at 3 days (**C** versus **D**) and 14 days (**K** versus **L**). All images were taken at ×40 magnification (inserts are additional ×2 magnification). Scale bar = 100 µm. Right panels (Q-T): numbers of Iba-1-positive microglia after sham occlusion (white columns) and UCO (grey columns). Three-way ANOVA including time, group and the three regions (IGWM1/2 and PVWM) as repeated measures showed a significant reduction in Iba1 positive microglia in the UCO groups within the parietal lobe regions (*P* < 0.05). Statistical significance shown on the figure was determined on individual time-points by one-way ANOVA for the peri-lesion region (^#^*P* < 0.05 sham versus UCO) and two-way ANOVA for the parietal lobe with area (IGWM1/2 and PVWM) as a repeated measure (*P < 0.05 sham versus UCO). Data are means ± SEM. N = Sham/UCO, Iba-1: 3d 9/8, 7d 8/8, 14d 9/8, 21d 9/8.

**Figure 6:**
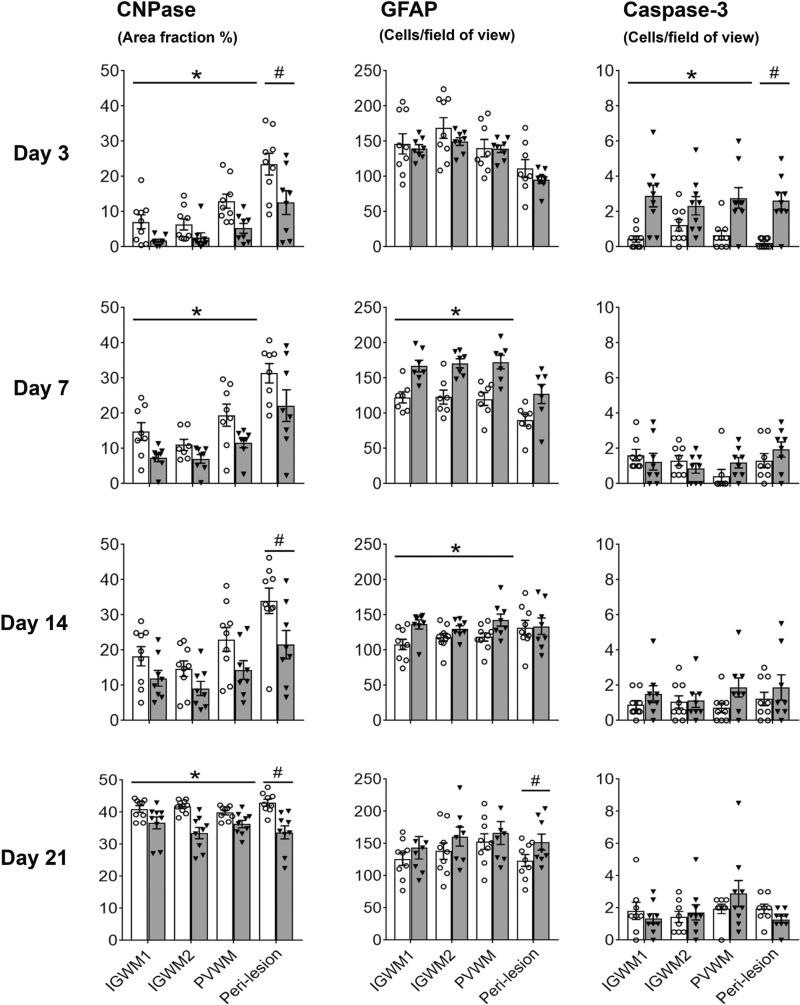
**Myelination, astrocytes and apoptotic cell death.** Graphs show area fraction of CNPase-positive myelin (left panels), numbers of GFAP positive astrocytes (middle panels) and numbers of activated-caspase-3 positive apoptotic cells (right panels) after sham occlusion (white columns) and UCO (grey columns). Three-way ANOVA including time and the three regions (IGWM1/2 and PVWM) as repeated measures showed a significant reduction in CNPase positive myelin and GFAP positive astrocytes in the UCO groups within the parietal lobe regions (*P* < 0.05). Two-way ANOVA including time as a repeated measure showed a significant reduction in CNPase-positive myelin in the UCO groups within the peri-lesion region of the temporal lobe (*P* < 0.05). Statistical significance shown in the figure was determined by one-way ANOVA for the peri-lesion region (^#^*P* < 0.05 sham versus UCO) and two-way ANOVA for the parietal lobe with area (IGWM1/2 and PVWM) as a repeated measure (*P < 0.05 sham versus UCO). Data are means ± SEM. N = Sham/UCO, CNPase: 3d 9/8, 7d 8/8, 14d 9/8, 21d 9/9. GFAP: 3d 9/8, 7d 7/7, 14d 9/8, 21d 9/8. Caspase-3: 3d 9/9, 7d 8/8, 14d 9/8, 21d 8/9.

In the peri-lesion region of the temporal lobe in both UCO and sham groups, there was a maturational increase over time for GFAP (*P* < 0.001), Iba-1 (*P* < 0.001), Olig-2 (*P* < 0.001) and CC1 (*P* < 0.001) cell counts, and for CNPase (*P* < 0.001) and MBP (*P* < 0.05) positive area fraction (Figs 2 and 4−6). UCO was associated with a significant reduction in CC1-positive cell counts (*P* < 0.001) and a significant reduction in CNPase (*P* < 0.001) and MBP-positive (*P* < 0.001) area fraction. There was no overall effect of UCO on GFAP, Iba-1, or Olig-2-positive cell counts (Figs 2, 5 and 6). *Posthoc* analysis in the peri-lesion region showed that UCO was associated with a significant reduction in Iba-1 positive cell counts in the 3d-UCO and 7d-UCO groups (both *P* < 0.05), a significant reduction in Olig-2 positive cell counts in the 21d-UCO group (*P* < 0.005) as well as a significant increase in Iba-1 (*P* < 0.05) and GFAP (*P* < 0.05) positive cell counts in the 21d-UCO group. Furthermore, there was a significant increase in caspase-3 positive cell counts in the 3d-UCO group (*P* < 0.05, Fig. 6). A trend towards a reduction in Olig-2-positive cell counts in the peri-lesion region was observed in the 3d-UCO group (*P* = 0.064).

### Milder diffuse WMI was observed in the parietal lobe

In contrast with the severe injury observed in the temporal lobe, the IGWM1/2 and PVWM regions of the parietal lobe showed only milder, diffuse WMI after UCO at all time-points (Figs 2 and 4 − 6). There was a maturational increase over time in Iba-1 (*P* < 0.001), Olig-2 (*P* < 0.001), and CC1-positive (*P* < 0.001) cell counts, and CNPase (*P* < 0.001) and MBP (*P* < 0.001) positive myelin area fraction within the IGWM1/2 and PVWM areas (Figs 2 and 4 − 6). UCO was associated with a significant reduction in CC1-positive cell counts (*P* < 0.05) in the IGWM1/2 and PVWM but no change in Olig-2-positive cell counts was observed (Fig. 2). UCO was associated with a significant reduction in Olig-2-positive cell count in the IGWM1/2 and PVWM regions in the 3d-UCO group (*P* < 0.05), but no differences were observed in the 7d-UCO, 14d-UCO and 21d-UCO groups (Fig. 2). UCO was associated with a small but significant increase in the numbers of caspase-3 positive cells in the IGWM1/2 and PVWM (*P* < 0.05), and a modest decrease in the area fraction of CNPase (*P* < 0.001, Fig. 6) and MBP (*P* < 0.05) positive myelination (Fig. 4) in these regions.

Morphologically, CNPase and MBP-positive myelination was markedly impaired in the IGWM1/2 and PVWM regions in the 3d-UCO and 7d-UCO groups including reduced density and fragmentation of myelin fibres. The 14d-UCO group showed a moderate reduction in density of myelin fibres. By contrast, myelination appeared to have recovered in the 21d-UCO group, with relatively intact morphology (Fig. 4). When the 21d groups were tested separately, the 21d-UCO group showed a persisting reduction in the area fraction of CNPase-positive myelination in the IGWM1/2 and PVWM (*P* < 0.005, Fig. 6) but no difference in the area fraction of MBP-positive myelination compared with the 21d-sham group (Fig. 4).

UCO was associated with increased numbers of Iba-1-positive (*P* < 0.001, Fig. 5) microglia and GFAP-positive (*P* < 0.05) astrocytes in the IGWM1/2 and PVWM at all time-points. The changes in microglial morphology at 3d after UCO in the IGWM1/2 and PVWM regions of the parietal lobe were similar to those observed in the temporal lobe, including large darkly labelled amoeboid microglia in the 3d-UCO group, with resolution towards sham morphology in the 7d-UCO group (Fig. 5). In contrast to that observed in the temporal lobe in the 14d-UCO and 21d-UCO groups, microglial morphology in the parietal IGWM1/2 and PVWM regions at these time-points appeared to show a higher proportion of ramified microglia.

**Table 1:** Fetal pH, blood gases and metabolites from the 21-day recovery groups

	Group	Baseline	UCO (5 min)	UCO (17 min)	+2 h	+4 h	+6 h	+1 day	+3 days	+7 days	+14 days	+21 days
pH	Sham	7.36 ± 0.01	7.36 ± 0.01	7.36 ± 0.01	7.36 ± 0.01	7.35 ± 0.01	7.35 ± 0.01	7.35 ± 0.01	7.34 ± 0.01	7.35 ± 0.01	7.34 ± 0.01	7.33 ± 0.01
	UCO	7.35 ± 0.01	7.03 ± 0.01^*^	6.84 ± 0.01^*^	7.29 ± 0.02^*^	7.38 ± 0.01	7.40 ± 0.01^*^	7.36 ± 0.01	7.36 ± 0.01	7.36 ± 0.01	7.35 ± 0.01	7.36 ± 0.01^*^
p_a_CO_2_ (mmHg)	Sham	48.5 ± 0.7	47.1 ± 0.8	46.6 ± 0.8	47.3 ± 0.9	46.1 ± 1.1	46.7 ± 0.9	48.4 ± 0.7	48.4 ± 0.8	49.1 ± 0.8	48.6 ± 1.0	49.4 ± 1.2
	UCO	50.7 ± 1.4	99.7 ± 2.6^*^	136.4 ± 6.0^*^	47.8 ± 0.8	47.7 ± 1.1	48.5 ± 0.7	46.3 ± 1.4	46.8 ± 1.5	49.2 ± 1.8	50.6 ± 1.4	48.6 ± 1.1
p_a_O_2_ (mmHg)	Sham	25.6 ± 0.6	24.6 ± 0.7	24.3 ± 0.6	25.5 ± 0.7	24.9 ± 0.5	25.7 ± 0.7	26.2 ± 0.8	27.0 ± 0.7	25.3 ± 1.1	26.0 ± 1.4	22.6 ± 1.3
	UCO	24.7 ± 0.8	6.6 ± 0.4^*^	9.5 ± 1.2^*^	27.2 ± 1.2	23.5 ± 1.0	25.4 ± 1.0	29.1 ± 1.0^*^	31.1 ± 1.2^*^	29.4 ± 1.5^*^	28.4 ± 1.2	26.5 ± 1.8
Hct (%)	Sham	27.2 ± 0.7	26.2 ± 0.8	26.1 ± 0.9	26.7 ± 0.7	25.4 ± 0.7	25.9 ± 0.9	26.9 ± 0.8	29.4 ± 1.4	31.6 ± 1.8	31.3 ± 1.3	31.4 ± 0.8
	UCO	26.2 ± 0.7	27.8 ± 1.1	28.2 ± 0.8	28.1 ± 0.7	27.4 ± 0.5^*^	27.7 ± 0.8	29.0 ± 0.5^*^	30.2 ± 2.2	33.6 ± 3.7	35.2 ± 4.4	30.5 ± 1.2
O_2_ct (mmol/l)	Sham	3.6 ± 0.1	3.4 ± 0.1	3.3 ± 0.2	3.6 ± 0.1	3.2 ± 0.1	3.4 ± 0.2	3.7 ± 0.2	3.9 ± 0.1	3.9 ± 0.2	6.6 ± 2.7	3.3 ± 0.3
	UCO	3.6 ± 0.1	0.4 ± 0.1^*^	0.5 ± 0.1^*^	4.3 ± 0.3^*^	3.6 ± 0.2	4.1 ± 0.2^*^	4.5 ± 0.1^*^	4.6 ± 0.2^*^	4.6 ± 0.2^*^	4.5 ± 0.2	4.2 ± 0.2^*^
Lactate (mmol/l)	Sham	0.9 ± 0.1	0.9 ± 0.03	0.9 ± 0.1	0.9 ± 0.0	0.9 ± 0.0	1.0 ± 0.0	0.9 ± 0.1	0.8 ± 0.04	0.9 ± 0.0	0.8 ± 0.0	1.1 ± 0.1
	UCO	0.8 ± 0.1	3.9 ± 0.2^*^	6.2 ± 0.3^*^	4.8 ± 0.7^*^	3.0 ± 0.5^*^	2.1 ± 0.2^*^	1.5 ± 0.2^*^	0.9 ± 0.1	0.7 ± 0.0^*^	0.8 ± 0.0	0.8 ± 0.0
Glucose (mmol/l)	Sham	1.1 ± 0.1	1.0 ± 0.04	1.0 ± 0.1	1.2 ± 0.1	1.1 ± 0.1	1.2 ± 0.1	1.1 ± 0.1	1.0 ± 0.1	1.0 ± 0.1	0.8 ± 0.1	0.8 ± 0.1
	UCO	1.0 ± 0.1	0.4 ± 0.1^*^	0.6 ± 0.1^*^	1.3 ± 0.1	1.3 ± 0.1	1.4 ± 0.1^*^	1.4 ± 0.1^*^	1.1 ± 0.1	0.9 ± 0.1	0.9 ± 0.1	0.9 ± 0.0

p_a_CO_2,_ arterial partial pressure of carbon dioxide; p_a_O_2,_ arterial partial pressure of oxygen; Hct, haematocrit; O_2_ct; arterial oxygen content.

* *P* < 0.05 sham versus UCO. Data are means ± SEM.

**Table 2:** Fetal demographics and post-mortem weights

Group	Sex F/M	Body weight (g)	Brain (g)	Heart (g)	Lungs (g)	Liver (g)	Kidneys (g)	Spleen (g)
3-Day Sham	5/4	1485.4 ± 50.7	29.2 ± 2.7	12.5 ± 0.5	54.5 ± 2.3	62.9 ± 2.7	7.2 ± 0.3	3.1 ± 0.2
3-Day UCO	5/4	1679.9 ± 105.6	25.2 ± 0.7^*^	12.3 ± 0.5	33.6 ± 1.5^*^	64.5 ± 8.4	7.3 ± 0.4	2.6 ± 0.2
7-Day Sham	3/5	1968.3 ± 180.2	34.3 ± 0.9	15.0 ± 0.9	60.3 ± 5.6	77.6 ± 6.1	7.9 ± 0.4	3.7 ± 0.4
7-Day UCO	4/4	2097.8 ± 159.5	27.4 ± 1.0^*^	14.3 ± 1.1	43.5 ± 7.5^*^	83.0 ± 6.3	7.3 ± 0.6	3.2 ± 0.3
14-Day Sham	6/3	2528.4 ± 110.9	33.4 ± 1.5	18.3 ± 0.7	81.3 ± 2.2	91.5 ± 8.0	8.9 ± 0.5	4.4 ± 0.2
14-Day UCO	6/2	2876.5 ± 310.2	28.2 ± 0.8^*^	19.4 ± 1.3	45.1 ± 3.7^*^	99.6 ± 14.8	10.2 ± 0.5	5.0 ± 0.5
21-Day Sham	4/5	3224.2 ± 204.3	39.5 ± 0.9	22.3 ± 0.8	88.9 ± 6.4	112.8 ± 10.4	12.4 ± 0.7	7.8 ± 0.9
21-Day UCO	6/3	3182.2 ± 157.4	32.5 ± 1.7^*^	20.8 ± 0.4	44.3 ± 5.6^*^	89.9 ± 6.8	11.3 ± 0.8	6.1 ± 0.5

* *P* < 0.05 sham versus UCO. Data are means ± SEM.

**Table 3:** Patterns of macroscopic WMI observed in the 21d-UCO group

Number of foetuses	Primary features	Secondary features
4	Marked white matter atrophy and ventriculomegaly	One foetus developed focal cystic white matter lesions in the temporal lobe and the second parasagittal gyrus of the parietal lobe
2	Extensive cystic white matter lesions in the temporal lobe	No ventriculomegaly
1	Focal cystic white matter lesions in the temporal lobe and first parasagittal gyrus of the parietal lobe	No ventriculomegaly or reduction in white matter area
2	No cystic lesions or ventriculomegaly	Moderate reduction in white matter area

## Discussion

The present study demonstrates that 3 and 7 days after HI both temporal and parietal lobe white matter show diffuse injury, characterized by selective cellular loss of mature oligodendrocytes, increased numbers of microglia and impaired MBP and CNPase positive myelination, similar to the typical pattern of diffuse non-cystic WMI in contemporary human post-mortem studies.^5^^,^^10^^,^^12^^,^^22^ In contrast, at 14 and 21 days after HI the temporal and parietal lobes showed very different patterns. Persistent diffuse WMI was observed in the parietal lobes, with evidence of partial repair of myelination despite continuing loss of mature oligodendrocytes. However in the temporal lobe, widespread tertiary cell death was seen by 14−21 days. At 21 days after HI, a spectrum of white matter degeneration was observed, including cystic-WMI in 4/9 foetuses or white matter atrophy with ventriculomegaly in an overlapping cohort of 4/9 foetuses; the 2/9 foetuses without severe WMI still had moderate loss of white matter area.

Cystic-WMI remains the most important risk factor for cerebral palsy among preterm infants,^10^ yet little is known about its aetiology or evolution.^6^ In the present study, the most severe injury was found in the white matter of the temporal lobe, including the majority of cystic lesions. Bilateral white matter lesions were found lateral to the sagittal strata, which remained intact, at the level of the trigone of the lateral ventricles. These regions are consistent with the inferior longitudinal fasciculus and overlying subcortical U fibres, which is a common site of cystic lesions in human preterm infants.^3^^,^^6^^,^^32^ The white matter lesions showed the classic appearance of cystic-WMI in preterm human infants, including the destruction of all cellular elements within the centre, infiltrating activated microglia and a dense border of severe astrogliosis and activated microglia surrounding the lesion, and disrupted myelination in the white matter surrounding the cystic lesions.^6^^,^^33^ In contrast, the parietal lobe showed cystic lesions in only two cases, but all animals showed persisting impairment of oligodendrocyte maturation, with reduced numbers of mature CC1 oligodendrocytes but sham levels of Olig-2 positive oligodendrocytes after 14 and 21 days recovery.

### Tertiary white matter cell death

UCO was associated with reduced brain weight at all time-points, and lateral ventricle area was increased in the 14d-UCO group suggesting that overall brain growth was impaired after UCO. Nevertheless, strikingly, there were no cystic lesions or reduction of white matter area in the 3d-UCO, 7d-UCO or 14d-UCO groups. It is notable that there was a maturational increase in cell numbers in the temporal lobe from 3 to 14 days. These findings suggest that widespread cell death leading to cystic necrosis and reduced white matter area must have occurred between 14 and 21 days after HI.

This very delayed evolution of cystic lesions in the present study is broadly consistent with the trajectory of cystic-WMI in humans, which typically take from 3 to 5 weeks to develop on serial cranial ultrasounds.^17^^,^^34^ Multiple studies in preterm infants have shown that cystic white matter lesions can eventually ‘disappear’, likely by collapsing, leading to ventriculomegaly.^11^^,^^17^ One study identified ventriculomegaly after the disappearance of cystic lesions in 15/78 infants, while 21/79 developed ventriculomegaly with cysts still visible.^17^ Overall, more severe and extensive cases of cystic-WMI evolved more rapidly.^17^ We may reasonably speculate from this evidence that the foetuses in the present study with marked ventriculomegaly had developed extensive white matter cystic lesions, which evolved more quickly and then collapsed before post-mortem.

### A potential role for excessive neuroinflammation after HI

The cystic white matter lesions at day 21 after UCO appeared to be preceded in the same regions by intense inflammation, including an early phase of microglial activation in the 3d-UCO group, and a second phase of intense microglial activation in the 14d-UCO group. Robust microglial activation is a hallmark of the early cellular response to perinatal HI.^35–37^ However, the longer-term changes in microglial activity after perinatal HI remain poorly understood.^38^ The second phase of microglial activation at 14d-UCO was most pronounced in white matter regions that harboured a microglial aggregate. These aggregates are transient but common developmental structures that form around the axonal crossroads at 19–24 gestational weeks in humans, contain large numbers of densely packed amoeboid microglia and are important in axonal guiding and pruning.^8^^,^^39^ Notably, the largest and most common aggregates were found in the temporal lobe at earlier time-points in both sham and UCO animals. Speculatively, pre-existing microglial aggregates at the time of HI may have provided an environment that predisposed white matter to cystic lesions.

The intense microglial changes in the 14d-UCO group suggest that delayed exacerbation of neuroinflammation may have been a critical mediator triggering tertiary cell death and macroscopic WMI in the present study. Understanding the temporal changes in microglial phenotypes may help determine the role of neuroinflammation in these findings. A limitation of the present study is that for technical reasons we are currently unable to identify microglial phenotypes in these tissues. Of interest, there is some evidence of delayed upregulation of pro-inflammatory microglial gene expression, up to 14 days after focal cerebral ischaemia in adult mice.^40^ Additionally, there is evidence in neonatal rats that delayed cystic grey matter injury can develop several weeks after moderate HI, in the Rice-Vannucci model.^41^

The mechanisms of this delayed cystic injury are unknown. There is considerable morphological evidence that early cell death after HI represents an apoptosis-necrosis ‘continuum’, with one or the other being more prominent depending on maturation, the nature of the insult and regional severity.^42^ Although these pathways overlap, apoptosis is unlikely to be major factor in cystic injury in the present study as there was little or no caspase-3 activity at times preceding tissue destruction. In contrast, programmed necrosis, which evolves slowly over weeks,^42^ is initiated by membrane-bound death receptor activation by the tumour necrosis factor family of cytokines.^43^ Potentially then, programmed necrosis may have been initiated in the tertiary phase by exuberant local neuroinflammation from microglial aggregates. Alternatively, it is possible that the biochemical cascades leading to programmed necrosis may have been initiated during HI, but took over 14 days to propagate and ultimately cause cell death.^42^^,^^44^

### *Persistent temporal lobe diffuse* WMI

Marked diffuse (non-cystic) WMI was also observed in regions of the temporal lobe that were spared from cystic lesions (i.e. in the peri-lesion region) in all nine foetuses in the 21d-UCO group. This was observed as a reduction in the numbers of both total and mature oligodendrocytes, morphologically abnormal myelination and reduced area fraction of CNPase and MBP-positive myelin. Diffuse WMI was associated with increased numbers of microglia, with a predominantly amoeboid morphology. The delayed reduction in total oligodendrocytes (Olig-2-positive) is striking considering the normal numbers of total oligodendrocytes at both 7 and 14 days after UCO, consistent with the exuberant proliferative response of oligodendrocyte progenitors after HI.^14^^,^^45^^,^^46^

### Recovery of parietal lobe myelination

In contrast with the temporal white matter, the IGWM1/2 and PVWM regions of the parietal lobe showed mild, diffuse WMI. There was an acute reduction in total Olig-2 positive oligodendrocytes in the 3d-UCO group, but this had fully recovered to sham values by 7 days.^14^^,^^45^^,^^46^ A persisting reduction in mature CC1 oligodendrocytes was observed at all time-points, consistent with oligodendrocyte maturational arrest, similar to previous reports.^12^^,^^19^^,^^45^ Strikingly, in the present study the morphology of both MBP and CNPase-positive myelination in the parietal lobe appeared to recover by 21 days after HI, with no reduction in the area fraction of MBP staining and only a small reduction in CNPase area fraction. Overall, these findings suggest that despite the delay in parietal lobe myelination after HI, the reduced number of mature oligodendrocytes was able to compensate and produce high levels of morphologically normal myelin by 21 days recovery. The IGWM and PVWM regions of the parietal lobe showed increased numbers of microglia, but in contrast to the temporal white matter, they predominantly showed a ramified morphology. Potentially, these microglia may provide a reparative function, and thereby contribute to recovery of myelination.

Finally, HI was associated with reduced lung weights at all time-points. We speculate that this may reflect impaired fetal breathing movements and subsequent impairment of lung growth.^47^

### Significance and perspectives

In the present study, we report delayed evolution of severe white matter degeneration after HI at preterm equivalent age, which broadly parallels the time course of severe, cystic-WMI in human preterm neonates.^17^^,^^34^ The majority of cell death after perinatal HI is often suggested to be largely finalized by 72 h, implying that only limited numbers of cells can be potentially salvaged by delayed therapeutic strategies.^15^^,^^48^^,^^49^ Strikingly, we now report that cystic-WMI was present 21 days after HI but not at 3, 7 or 14 days, suggesting the tantalizing possibility that substantial white matter protection may be possible with even very delayed interventions. Speculatively, the spatial relationship between the distribution of microglial aggregates at the time of HI and subsequent cystic necrosis suggests that delayed inflammatory responses may be a key determinant of the localization of cystic white matter lesions and thus that anti-inflammatory interventions may be beneficial. Further pragmatic studies are needed to clarify when cells irreversibly commit to delayed cell death in the tertiary phase.

## Abbreviations

CC1 =: anti-adenomatous polyposis coli
CNPase =: 2',3'-cyclic-nucleotide 3'-phosphodiesterase
GFAP =: glial fibrillary acidic protein
Hct =: haematocrit
HI =: hypoxia-ischaemia
Iba-1 =: ionized calcium binding adaptor molecule-1
IGWM =: intragyral white matter
MAP =: mean arterial pressure
MBP =: myelin basic protein
O_2_ct =: arterial oxygen content
Olig-2 =: oligodendrocyte transcription factor-2
p_a_CO_2_ =: arterial partial pressure of carbon dioxide
p_a_O_2_ =: arterial partial pressure of oxygen
PVWM =: periventricular white matter
SEM =: standard error of the mean
TNF =: tumour necrosis factor
UCO =: umbilical cord occlusion
WMI =: white matter injury
